# Avian Influenza a H9N2 Viruses in Morocco, 2018–2019

**DOI:** 10.3390/v14030529

**Published:** 2022-03-04

**Authors:** Fatima-Zohra Sikht, Mariette Ducatez, Charifa Drissi Touzani, Adam Rubrum, Richard Webby, Mohammed El Houadfi, Nour-Said Tligui, Christelle Camus, Siham Fellahi

**Affiliations:** 1 Avian Pathology Unit, Department of Veterinary Pathology and Public Health, Agronomy and Veterinary Institute Hassan II, Rabat B.P. 6202, Morocco; fz.sikht@iav.ac.ma (F.-Z.S.); charifadrissi@gmail.com (C.D.T.); elhouadfimohammed@yahoo.fr (M.E.H.); fellahisiham2015@gmail.com (S.F.); 2 IHAP, Toulouse University, INRAE, ENVT, 31300 Toulouse, France; mariette.ducatez@envt.fr; 3 Department of Infectious Diseases, St. Jude Children’s Research Hospital, Memphis, TN 38105, USA; adam.rubrum@stjude.org (A.R.); richard.webby@stjude.org (R.W.); 4 Anatomo-Pathology Unit, Department of Veterinary Pathology and Public Health, Agronomy and Veterinary Institute Hassan II, Rabat B.P. 6202, Morocco; n.tligui@iav.ac.ma

**Keywords:** low pathogenic avian influenza virus, H9N2, Morocco, sequencing, full genome

## Abstract

Low pathogenic H9N2 avian influenza (LPAI H9N2) is considered one of the most important diseases found in poultry (broiler, laying hens, breeding chickens, and turkeys). This infection causes considerable economic losses. The objective of this work was to monitor and assess the presence of avian influenza virus (AIV) H9N2 in eight different regions of Morocco using real-time RT-PCR, and to assess the phylogenetic and molecular evolution of the H9N2 viruses between 2016 and 2019. Field samples were collected from 108 farms suspected of being infected with LPAI H9N2 virus. Samples were analyzed using H9N2-specific real-time RT-PCR. Highly positive samples were subjected to virus isolation and seven isolates were fully sequenced. Low pathogenic H9N2 avian influenza virus was introduced in Morocco in 2016. We show that in 2018–2019, the virus was still present irrespective of vaccination status. Phylogenetic and molecular analyses showed mutations related to virulence, although our viruses were related to 2016 Moroccan viruses and grouped in the G1 lineage. Specific amino acid substitutions were identified in Moroccan H9N2 viruses that are believed to lead to increased resistance to antiviral drugs.

## 1. Introduction

Low pathogenic H9N2 avian influenza is an emerging disease that causes important economic losses in the poultry sector and is considered a threat to both poultry farms and public health.

Being a member of the genus *Alphainfluenzavirus*, and of the family *Orthomyxoviridae*, avian influenza viruses are enveloped RNA virus, with a genome composed of eight negative-sense RNA segments [1]. They are classified as low or highly pathogenic, on the basis of their virulence and hemagglutinin (HA) and neuraminidase (NA) sequences [2]. There are currently18 HA and 11 NA identified, including the bat-specific H17–H18 and N10–N11 [3]. Avian influenza virus subtype H9N2 is pathotyped as a low pathogenic virus (LPAI). However, co-infections with other pathogens can lead to severe outbreaks with high mortality rates and severe economic losses [4,5].

H9N2 LPAIV had first been described in 1966 in a turkey farm in the USA [6]. Since then, it has been reported in numerous countries around the world. Between 1992 and 1994, an H9N2 outbreak occurred in Guangdong Province, China, and affected chicken farms with a mortality rate of 10% to 40%, with a reduction in egg-laying rate of 14% to 75% [7].

In 1996, H9N2 LPAIV was reported in South Korea [8]. In 1998, it was isolated from most provinces in China and, as a result, it was considered to be one of the most widespread avian influenza virus in China [9]. Other countries in the Middle East and North Africa have been affected by this virus including Iran [10], Saudi Arabia [11], Jordan [12], the United Arab Emirates [13], Tunisia [14], Egypt [15], Sultanate of Oman [16], and Libya [17].

Phylogenetic analysis of the genome of LPAI H9N2 strains allowed to classify this virus into two distinct lineages: Eurasian and American. Though the Eurasian lineage contains several clades, most of the strains detected were classified in two clades (G1 and Y280) [18]. The G1 clade is represented by the A/Quail/Hong Kong/G1/1997 prototype virus, which mainly circulates in South China, Central Asia and the Middle East, while the Y280 clade viruses circulate throughout China and are represented by the A/Duck/Hong Kong/Y280/1997 prototype.

The main sources of LPAI H9N2 infections are the domestic and wild avian species. Wild birds are considered one of the reservoirs of the virus, and can transmit it over long distances. Transmission of the H9N2 virus can occur through direct contact with infected animals, and the infection can spread between farms through the movement of infected birds, vehicles, contaminated equipment or people with contaminated shoes or clothing [19]. In Pakistan, sparrows were shown to play a very important role in the transmission of the virus between farms [20]. In general, the sensitivity and receptivity of H9N2 is strongly dependent on the avian species (chicken and turkey). However, other species raised for consumption and/or hunting, such as guinea fowl, quail, pheasant, partridge, duck, goose, and ostrich are also considered sensitive. LPAI H9N2 virus has also been reported to be transmissible to mammals including dogs and cats [21] as well as humans [22,23,24,25].

The LPAIV H9N2 strain (SF1, GenBank accession number SCA48100) introduced in Morocco in January 2016, belongs to the G1 lineage, and is closely related to viruses circulating in the Middle East. As a response, the competent authorities authorized vaccination of any type of chicken as the best choice to limit the rapid spread of this disease [26]. However, in order to guide veterinarians to a rational choice of vaccines, it is important to determine and to phylogenetically analyze the circulating strains.

The aim of this study was to monitor the presence of LPAI H9N2 viruses in farms where animals with respiratory signs are reported using real-time reverse transcription PCR. Sequencing of isolates was performed in order to detect potential mutations that might affect the efficacy of commercial vaccines.

## 2. Materials and Methods

### 2.1. Specimen Collection

In collaboration with private veterinarians, a total of 151 samples, which included organs (trachea, lungs) and tracheal swabs, were collected from 108 commercial broilers farms (vaccinated and non-vaccinated) in eight regions of Morocco. Our sampling was based on chickens suspected of being infected with LPAI H9N2 virus, and presenting respiratory signs (rales, sneezing), associated with a decrease in food consumption and a drop in production. The specimens were collected in a period of 11 months, from 28/06/2018 to 31/05/2019.

### 2.2. Samples Processing

#### 2.2.1. RNA Extraction and Real Time RT-PCR

RNA extraction was performed using the NucleoSpin^®^ RNA Virus Kit (Macherey-Nagel, Düren, Germany, No. 740956.250), following the manufacturer’s instructions. In order to detect the H9N2 virus, the extracted RNA was amplified on the 7500 Fast Real-Time PCR System thermal cycler (Applied Biosystems, Foster City, CA, USA), using the primers and probe for generic detection of H9 subtypes described by [27], which target a conserved region in the HA2 subunit of the HA gene sequence.

#### 2.2.2. Virus Isolation

In order to obtain a maximum viral load detectable by conventional RT-PCR and for full genome sequencing purposes, 17 samples among those with the highest Ct in RT-qPCR, from 17 different farms, were grown on 10-day-old, specific pathogen free (SPF) embryonated eggs. Briefly, the eggs were mirrored and the air chamber was delimited. The viral inoculums were prepared by mixing 0.2 mL of the viral suspension, 0.6 mL of sterile PBS and 0.2 mL of antibiotic OXY-Kel 20 L.A (oxytetracycline) and injected via allantoic cavity route using a sterile needle into the air chamber of the embryonated eggs. After viral inoculation, the eggs were incubated at 37 °C and examined daily for five days to assess the viability of the embryos. After the death of the embryo, eggs were refrigerated at 4 °C for 4 h. Then, the lesions on the embryos were observed and the allantoic fluids were collected, clarified, and stored at −80 °C until use.

#### 2.2.3. Full Genome Amplification and Sequencing of H9N2 Moroccan Isolates

Viral RNA was extracted from allantoic fluids harvested from the 7 SPF embryonated eggs with the highest viral load, using the Macherey Nagel kit (Duren, Germany, No. 740956.250). Whole genome sequencing of 2018–2019 Moroccan isolates was performed with an Illumina MiSeq System (Illumina, San Diego, CA, USA) as previously described [28]. The preparation of libraries was performed using an Illumina Nextera XT library prep kit (FC-131-1096) (Illumina, San Diego, CA, USA) following the manufacturer’s instructions. A tape station was used to verify the library quantity and quality. CLC genomic workbench was used for genomes assembly. The nucleotides sequences of all characterized strains in this study are submitted in the GenBank database under accession numbers summarized in Table 1.

### 2.3. Sequences and Phylogenetic Analyses

Bioedit 7.2.5 software [29] and ClustalW (version 1.83) [30] were used to compare and align nucleotide sequences of the complete genomes of seven Moroccan H9N2 isolates.

The phylogenetic tree was constructed by the maximum likelihood method, using the Mega 6.06 software [31]. The Blast [32] and Bioedit programs [30] were used to determine the sequence identity and compare the Moroccan strains with those selected from Genbank.

### 2.4. Statistical Analysis

Statistics describing the correlation between H9N2 positivity and the different factors: regions and vaccination status were calculated for each variable, including the mean and percentage distribution of frequencies. A non-parametric test (chi-squared test) was used to calculate the correlation between the H9N2 frequency in farms and their vaccination status.

## 3. Results

### 3.1. Case History and H9N2 Detection

One hundred and fifty-one samples from respiratory tissues and tracheal swabs were collected between June 2018 and May 2019 from different areas of Morocco: Fes-Meknes, Rabat-Sale-Kenitra, Casablanca-Settat, Draa-Tafilalet, Benimellal-Khenifra, Souss-Massa, Marrakech-Safi, and the eastern region. The samples were tested by real time RT-PCR to detect the presence of influenza virus. A total of 83 were positive for AIV with cycle threshold (Ct) values varying from 12 to 39 (Table A1), of which 40%, 56%, and 4% of the samples had a Ct below or equal to 25, between 25 and 35, and above 35, respectively. The epidemiological survey resulted in a positivity rate of the disease of 58% (63 positive farms out of 108 sampled farms) (Table 2).

### 3.2. Vaccination Status

The positivity rate (relative prevalence) of LPAI H9N2 positive farms was estimated to be 50% in unvaccinated farms (32 positive farms out of 64 chicken unvaccinated farms tested), while it was 70% in vaccinated farms (31 positive farms out of 44 chicken vaccinated farms tested). The overall vaccination rate reached 41% (44 out of 108 farms tested against H9N2) (Table 2).

The presence of LPAIV H9N2 was detected differently between the groups of vaccinated and unvaccinated farms, but the difference was not statistically significant (95% CI, *p* value: 0.9).

### 3.3. Molecular Characterization and Phylogenetic Analysis of the Eight Viral Segments

Viruses from highly positive samples were isolated. The genome of 7 of them was fully sequenced with an IlluminaMiSeq System [28]. Phylogenetic analysis showed that our Moroccan H9N2 viruses isolated from chickens were in the same cluster as the other Moroccan viruses detected in 2016, and grouped into G1 lineage. They were compared with relevant virus sequences available in GenBank.

**Figure 1:**
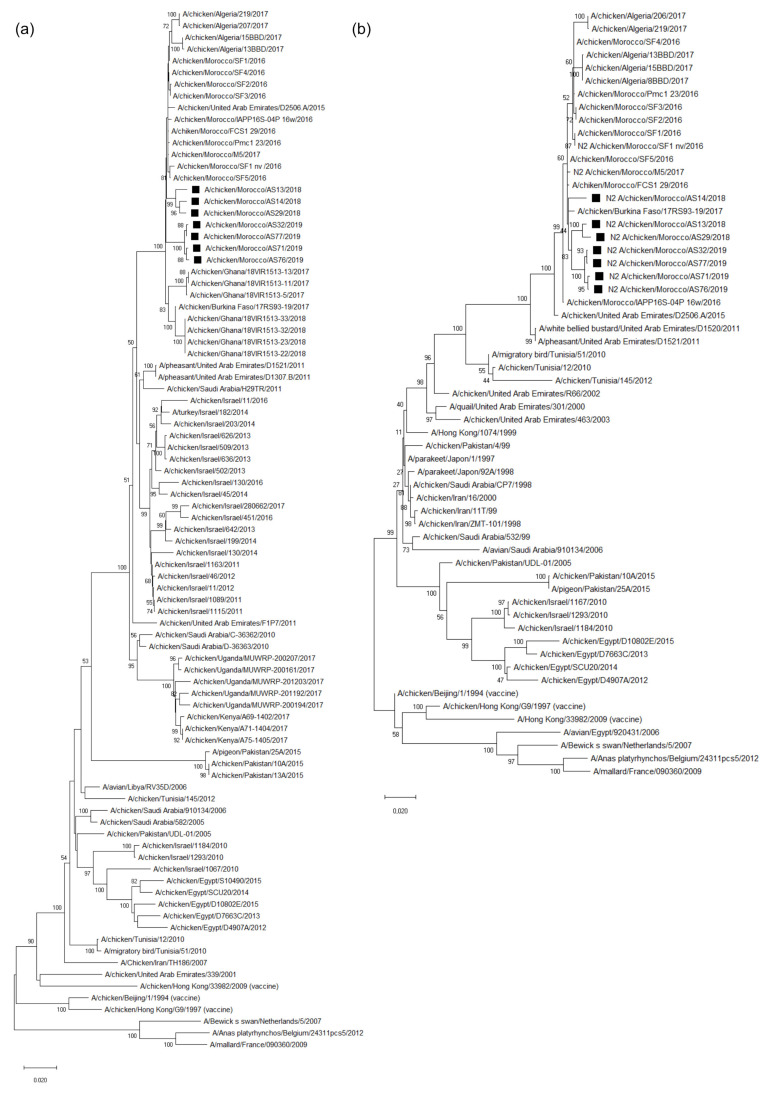
Phylogenetic trees of Moroccan HA (**a**) and NA (**b**) gene segments. The nucleotide sequences of Moroccan H9N2 viruses (black squares) characterized in this study were compared with relevant virus sequences available in GenBank and GISAID databases, reference viruses, and relevant sequences from neighboring areas.

**Figure A1:**
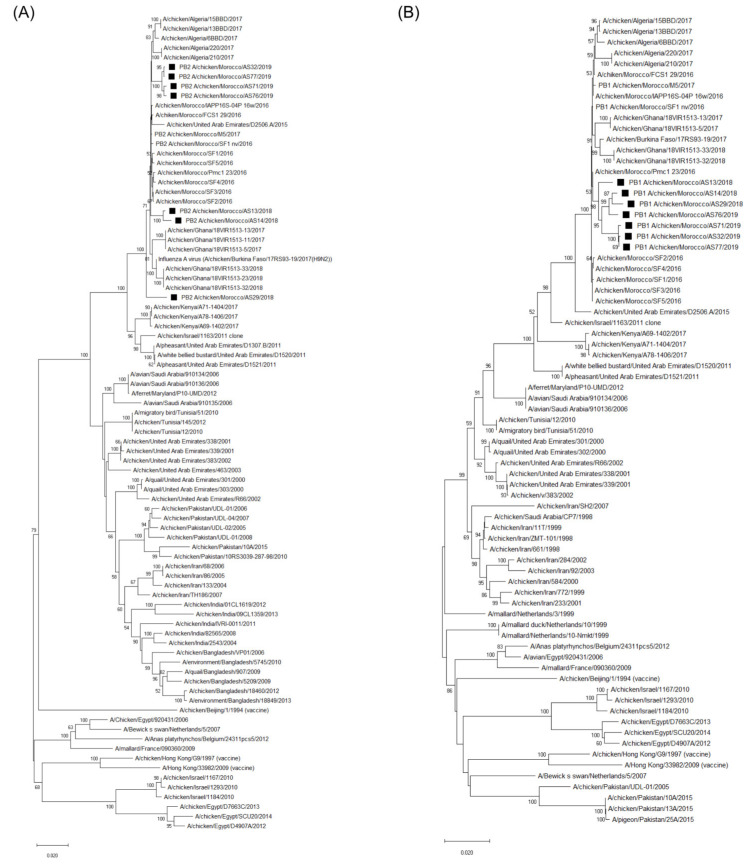

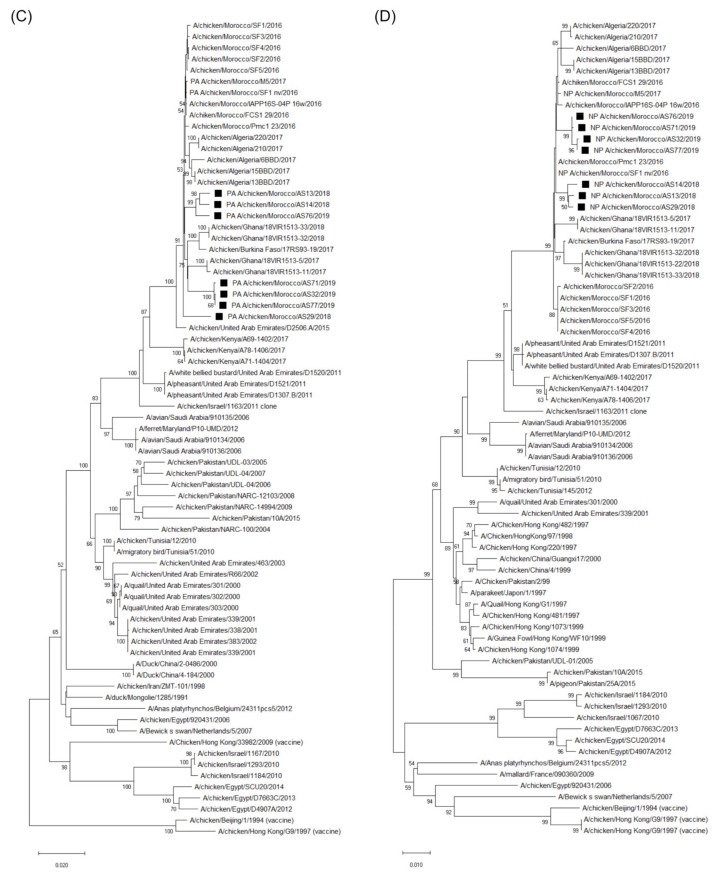

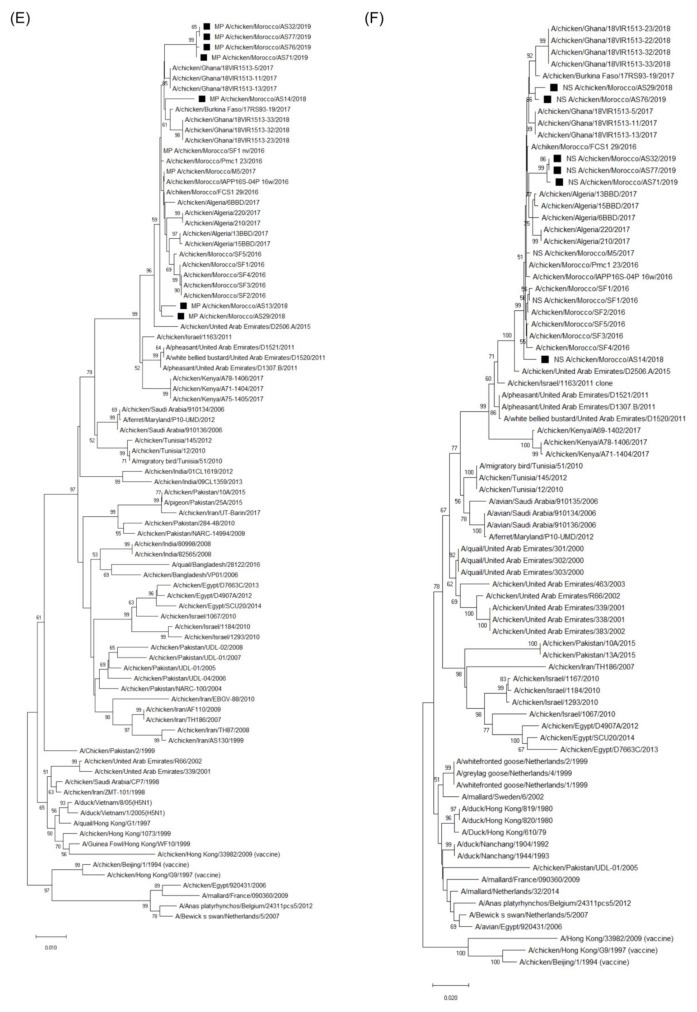
Phylogenetic trees of Moroccan PB2 (**A**), PB1 (**B**), PA (**C**), NP (**D**), M (**E**), and NS (**F**) segments. The nucleotide sequences of Moroccan H9N2 viruses characterized in this study (black squares) were compared with relevant virus sequences available in GenBank and GISAID databases, reference viruses, and relevant sequences from neighboring areas.

Based on HA and NA phylogenetic trees, our Moroccan viruses were closely related to viruses previously isolated in the Emirates (2015), Morocco (2016), Burkina Faso (2017), and Algeria (2017), with bootstrap values of 100 and 60 for HA and NA, respectively (Figure 1). Regarding the internal genes, they grouped with the Moroccan viruses of 2016–2017, Algerian viruses of 2017, and Ghana viruses of 2017–2018 (Figure A1).

The sequence analyses of the seven Moroccan isolates showed several substitutions in both HA and NA sequences when compared to 2016 strain SF1 (Table A2 and Table A3).

All seven Moroccan isolates had the RSSR*GLF motif at the HA cleavage site, which is a characteristic and signature of the low pathogenic H9N2 viruses.

Potential HA glycosylation sites were identical to 2016 Moroccan viruses (29 NSTE, 82 NPSC, 105 NGTC, 141 NVTY, 298 NSTM, 492 NGTY, H3 numbering throughout), except for position site (297 NISK→NVSK) for four out of seven samples.

Our viruses did not present HA Receptor Binding Site (RBS) sequence associated with greater affinity for 6′-sialylacetyllactosamine (6SLN) [33] (Table 3), nor, when compared to Moroccan 2016 viruses, new critical amino acids defined as supporting mammalian replication [34,35,36].

Among mutations associated to resistance to antiviral molecules, no changes from SF1 strain were identified for NA H274Y substitution [37] or M2 S31N mutation [38] (Table 3).

## 4. Discussion

Our analysis included 151 field samples from 108 poultry farms suspected of being infected by the LPAI H9N2 virus and presenting mainly respiratory signs as well as poor zootechnical performances (decrease in production, decrease in feed consumption and mortalities), as reported previously [39].

The results revealed that 58% of the samples were positive for LPAIV H9N2. However, we cannot extrapolate these results on the epidemiological profile of the LPAI H9N2 virus in Morocco since our sampling was not representative enough of the national territory and only 8 regions out of 12 (Fes-Meknes, Rabat-Sale-Kenitra, Casablanca-Settat, Draa-Tafilalet, Béni Mellal-Khenifra, Souss-Massa, Marrakech-Safi and the Oriental) were sampled.

The spread of LPAI H9N2 virus in Morocco can be explained mainly by the movement of farmers, workers, and feed suppliers without compliance with biosecurity rules, to which can be added the transport of live chickens [13,15,26]. It should be noted that the application of sanitary biosecurity measures in broiler farms has been shown to be insufficient to prevent the entry of the virus [26]. In addition, mutations associated with resistance to antiviral molecules are still present in our LPAI H9N2 strains. The M2 S31N mutation is known to increase resistance to antiviral molecules, especially amantadine and rimantadine [38]. Likewise, other studies have shown that the absence of the H274Y substitution in the NA protein can confer to the virus a sensitivity to neuraminidase inhibitors such as oseltamivir [37].

The positivity rate of AI H9N2 positive farms was estimated to be 50% in unvaccinated chicken farms (32 positive farms out of 64 chicken-unvaccinated farms tested), while it reached 70% in vaccinated chicken farms (31 positive farms out of 44 chicken-vaccinated farms tested). A recent study evaluating the efficacy of four different commercial vaccines on H9N2 LPAIV SF1 strain has shown that they conferred a very limited protection against the infection [40]. Park and collaborators indicated that vaccination against H9N2 virus coupled with continuous infection of vaccinated flocks is an advantage for mutant viruses selection [41], whereas other studies report that vaccination decreases viral pressure in the field by reducing the level and duration of viral shedding [42]. Other explanations include the quality of the vaccine (either that it was not of the same strain as the virus currently circulating, or that it had a low antigen concentration [43,44]). We were not able to gather sufficient information relative to the vaccines used and their composition to be able to conclude on this point.

In addition, the vaccination rate against H9N2 was low (41%), which can be explained by the high cost of vaccination or by the fact that some farmers consider the vaccine is ineffective.

For unvaccinated specimens that tested negative, despite respiratory signs, other respiratory diseases, including BI or NDV, might be the cause of the observed clinical signs.

In this study, we demonstrated a relationship between our viruses, isolated in 2018–2019, and those isolated in Morocco in 2016, which all belong to the G1 lineage [26]. In order to evaluate the evolution of the Moroccan H9N2 virus over time (i.e., after its first introduction into Morocco), phylogenetic and genetic analyses were carried out.

On the HA and NA phylogeny, the 2018–2019 Moroccan viruses were close to those from Emirates (2015), Morocco (2016), Burkina Faso (2017), and Algeria (2017). As for internal genes, they were grouped in the same cluster as the Moroccan viruses of 2016–2017, Algerian viruses of 2017, and Ghana viruses of 2017–2018. This similarity can be explained by the common border between Morocco and Algeria, and by the history of commercial exchanges within western Africa countries. The evolution of the influenza virus directly depends on its genomic properties, which leads us to follow and verify the presence of possible mutations over time (especially on the HA and NA genes, which are the main proteins targeted by antibodies). Moroccan viruses harbor several mutations in HA and NA. Some have already been characterized, such as HA Q226L, which is known to enhance binding to mammalian-like receptors [45]. For other mutations, further studies are necessary in order to determine whether they could affect the virulence of the virus in poultry, or increase transmissibility to human. Potential glycosylation sites were identified in our Moroccan strains. As compared to Moroccan strains isolated in 2016, there was one amino acid change within a glycosylation site (297 NISK→NVSK, H3 numbering throughout) in four of the seven sequenced isolates. Changes in glycosylation sites may affect the host range and virulence of influenza viruses [46], though we do not know if it is the case here.

## 5. Conclusions

The low pathogenic avian influenza virus H9N2 is endemic within the country despite vaccination. Biosecurity issues in farm management, combined with high mutation potential are likely to cause dynamic changes in LPAI H9N2 strains. This prompts us to propose appropriate surveillance and adaptation of vaccines to circulating strains in order to better understand and fight public health risks.

## Figures and Tables

**Table 1:** Accession numbers of segments sequences of studied Moroccan H9N2 viruses.

Segment	Strain						
	AS13	AS14	AS29	AS32	AS71	AS76	AS77
PB2	MW165151	MW165079	MW165121	MW165089	MW165136	MW165110	MW165106
PB1	MW165154	MW165125	MW165122	MW165088	MW165142	MW165113	MW165101
PA	MW165158	MW165082	MW165117	MW165085	MW165139	MW165116	MW165103
HA	MW165152	MW165084	MW165120	MW165090	MW165137	MW165111	MW165105
NP	MW165157	MW165083	MW165124	MW165086	MW165140	MW165109	MW165108
NA	MW165155	MW165078	MW165119	MW165092	MW165135	MW165115	MW165104
NS	MW165156	MW165080	MW165123	MW165091	MW165141	MW165114	MW165102
M	MW165153	MW165081	MW165118	MW165087	MW165138	MW165112	MW165107

AS13, A/chicken/Morocco/AS13/2018; AS14, A/chicken/Morocco/AS14/2018; AS29, A/chicken/Morocco/AS29/2018; AS32, A/chicken/Morocco/AS32/2019; AS71, A/chicken/Morocco/AS71/2019; AS76, A/chicken/Morocco/AS76/2019; AS77, A/chicken/Morocco/AS77/2019.

**Table 2:** Farms positivity rate.

	Number of Farms	Positive Farms	Positivity Rate
Fes-Meknes	34	20	59%
Rabat-Sale-Kenitra	18	8	44%
Casablanca-Settat	16	13	81%
Draa-Tafilalet	1	1	100%
BeniMellal-Khenifra	4	3	75%
Souss-Massa	26	17	65%
Oriental	4	1	25%
Marrakech-Safi	5	0	0%
Morocco (Total)	108	63	58%
Vaccinated	44 (41%)	31	70%
Unvaccinated	64 (59%)	32	50%

**Table 3:** Residues associated to 6′-sialylacetyllactosamine-affinity and to drug resistance.

HA * RBS				NA	M2
	190	Q226L	227	274	S31N
SF1 **	V	L	I	Q	N
AS13	T	L	I	Q	N
AS14	A	L	I	Q	N
AS29	V	L	I	Q	N
AS32	A	L	I	Q	N
AS71	A	L	I	Q	N
AS76	A	Q	I	Q	N
AS77	A	Q	I	Q	N

HA, hemagglutinin; RBS, receptor-binding site. * H3 numbering; ** GenBank accession number: SCA48100.

## Data Availability

Not applicable.

## Appendix A

**Table A1:** List and status of samples used in this study.

Sample	Farm	Sampling Date	Location of Farm	Age of Birds	H9N2 Vaccination Status	H9N2 RT-qPCRCt Value	Farms Status
AS1	F1	28/06/2018	Rabat	27D	V	25	P
AS2	F2	16/07/2018	Rabat	46D	V	-	N
AS3				46D		-	
AS4				46D		-	
AS5	F3	08/08/2018	Oriental	45D	NV	-	N
AS6				45D		-	
AS7	F4	08/08/2018	Oriental	36D	NV	-	N
AS8	F5	17/07/2018	Casablanca	28D	V	-	N
AS9	F6	17/09/2018	Temara	43D	V	-	N
AS11	F7	19/10/2018	Meknes	32D	NV	-	N
AS12B1 *	F8	23/10/2018	Kenitra	23D	NV	20	P
AS12B2		23/10/2018		23D		33	
AS13 *^+^	F9	18/10/2018	El hajeb	32D	NV	17	P
AS14 *^+^	F10	24/10/2018	Meknes	30D	NV	18	P
AS15	F11	21/10/2018	Meknes	37D	NV	-	N
AS16 *	F12	17/10/2018	Midelt	40D	NV	18	P
AS17	F13	11/12/2018	Fes	38D	NV	26	P
AS18	F14	11/12/2018	Fes	35D	NV	26	P
AS19	F15	11/12/2018	Fes	41D	NV	-	N
AS20	F16	23/11/2018	Fes	32D	V	25	P
AS21	F17	12/11/2018	Salé	36D	V	-	N
AS22	F18	07/12/2019	Meknes	34D	NV	22	P
AS23		07/12/2019		34D		26	
AS24		07/12/2019		34D		22	
AS26	F19	19/02/2019	Benslimane	30D	NV	-	N
AS27	F20	20/02/2019	Rabat	24D	NV	-	N
AAS28	F21	04/11/2018	Ait brahim (Fes)	32D	V	24	P
AS29 *^+^	F22	16/11/2018	Fes	36D	V	16	P
AS30	F23	26/01/2019	Hajeb	20D	V	24	P
AS31	F24	11/02/2019	Sefrou	30–36D	V	17	P
AS32 *^+^	F25	12/02/2019	Ain chegag	37D	V	19	P
AS33 *	F26	13/02/2019	Zerarda tahla	40D	NV	16	P
AS34	F27	25/01/2019	Khemisset	33D	NV	-	N
AS35	F28	04/03/2019	Meknes	32D		-	N
AS36	F29	13/02/2019	Fes	34D		14	P
AS37	F30	13/02/2019	Fes	37D	V	-	N
AS38	F31	01/02/2019	Meknes	32D	NV	-	N
AS39	F32	12/02/2019	Hadeb	34D	NV	-	N
AS40	F33	12/02/2019	Salé	42D	NV	12	P
AS41	F34	25/02/2019	Khemisset	44D	V	-	N
AS42	F35	25/02/2019	Meknes	35D	NV	-	N
AS43 *	F36	07/02/2019	Casablanca	29D	V	16	P
AS44	F37	07/02/2019	Casablanca	32D	V	16	P
AS45 *	F38	10/02/2019	Rabat	45D	V	17	P
AS46		10/02/2019		45D		14	
AS47	F39	11/02/2019	Tiflet	28D	NV	-	N
AS54	F40	20/02/2019	Rabat	32D	V	24	P
AS55	F41	20/02/2019	Oriental	37D	NV	-	N
AS56 *	F42	20/02/2019	Oriental	38D	NV	26	P
AS57	F43	21/02/2019	Taza	29D	NV	26	P
AS58	F44	21/02/2019	Tahla	36D	NV	-	P
AS59		21/02/2019		36D		26	
AS60	F45	21/02/2019	Tahla	39D	NV	-	N
AS61 *	F46	24/02/2019	Meknes	44D	NV	21	P
AS62	F47	24/02/2019	Elhajeb	37D	NV	23	P
AS63		24/02/2019		37D		12	
AS64	F48	24/02/2019	Elhajeb	35D	NV	26	P
AS65	F49	25/02/2019	Meknes	41D	NV	-	N
AS66	F50	25/02/2019	Meknes	35D	NV	-	N
AS67	F51	25/02/2019	Meknes	32D	NV	-	N
AS68 *	F52	26/02/2019	Khenifra	38D	NV	14	P
AS69	F53	25/02/2019	Khenifra	28D	NV	25	P
AS70	F54	28/02/2019	Salé	23D	NV	22	P
AS71 *^+^	F55	01/04/2019	Sidi slimane	14D	NV	22	P
AS72	F56	11/12/2017	Khenifra	33D	NV	-	N
AS73	F57	20/02/2019	Khenifra	36D	NV	14	P
AS74	F58	11/04/2019	Sidi slimane	37D	NV	-	N
AS75	F59	06/03/2019	Salé	29D	NV	-	N
AS76 *^+^	F60	23/02/2019	Meknes	38D	NV	20	P
AS77 *^+^	F61	17/01/2019	Meknes	29D	NV	16	P
AS78	F62	01/04/2019	Tiznit	25D	NV	-	P
AS79		01/04/2019		25D	NV	30	
AS80	F63	01/04/2019	Tiznit	32D	NV	30	P
AS81		01/04/2019		32D	NV	28	
AS82	F64	01/04/2019	Tiznit	32D	NV	-	N
AS83		01/04/2019		37D	NV	-	
AS84	F65	01/04/2019	Tiznit	37D	NV	-	N
AS85 *	F66	01/04/2019	Tiznit	28D	NV	16	P
AS86	F67	01/04/2019	Tiznit	37D	NV	-	N
AS87		01/04/2019		34D	NV	-	
AS88	F68	01/04/2019	Tiznit	34D	NV	-	N
AS89	F69	01/04/2019	Tiznit	15D	NV	26	P
AS90	F70	31/05/2019	Rabat	32D	NV	-	N
AS91		31/05/2019		32D		-	
AS92		31/05/2019		32D		-	
AS93		31/05/2019		32D		-	
AS94		31/05/2019		32D		-	
AS95		31/05/2019		32D		-	
AS96		31/05/2019		32D		-	
AS97		31/05/2019		32D		-	
AS98		31/05/2019		32D		-	
BS1	F71	09/04/2019	Fes	28D	NV	-	N
BS2	F72	09/04/2019	Fes	36D	NV	-	N
BS3	F73	16/09/2019	Ait moussa	35D	NV	-	N
BS4		16/09/2019		35D		-	
BS5	F74	08/10/2019	Ait moussa	36D	V	-	N
BS6		09/10/2019		36D		-	
BS7	F75	23/10/2019	Marrakech	18D	NV	-	N
BS8	F76	24/10/2019	Haouz	29D	NV	-	N
BS9	F77	30/10/2019	Marrakech	34D	V	-	N
BS10	F78	31/10/2019	Rhamna	13D	NV	-	N
BS11	F79	05/11/2019	Marrakech	12D	NV	-	N
BS12	F80	23/11/2019	Casablanca	30D	NV	29	P
BS13	F81	23/11/2019	Casablanca	21D	NV	28	P
BS14	F82	23/11/2019	Casablanca	24D	NV	33	P
BS15	F83	23/11/2019	Casablanca	24D	NV	31	P
BS18	F84	25/11/2019	Tiznit	34D	V	-	N
BS19		25/11/2019		34D		-	
BS20		25/11/2019		34D		-	
BS21	F85	05/12/2019	Tiznit	29D	V	-	N
BS22		05/12/2019		29D		-	
BS23		05/12/2019		29D		-	
BS47	F86	14/11/2019	Casablanca	28D	V	26	P
BS48	F87	14/11/2019	Casablanca	28D	V	26	P
BS49	F88	14/11/2019	Casablanca	28D	V	27	P
BS50	F89	24/10/2019	Mohammedia	28D	V	-	N
BS57	F90	05/03/2020	Casablanca	34D	V	29	P
BS58		05/03/2020		34D		-	
BS59	F91	05/03/2020	Casablanca	30D	V	-	P
BS60		05/03/2020		30D		33	
BS61	F92	02/07/2020	Tiznit	42D	V	-	N
BS62	F93	02/07/2020	Tiznit	34D	V	30	P
BS63	F94	02/07/2020	Tiznit	30D	V	30	P
BS64	F95	02/07/2020	Tiznit	22D	V	30	P
BS65	F96	02/07/2020	Tiznit	30D	V	29	P
BS66	F97	02/07/2020	Tiznit	35D	V	32	P
BS67	F98	02/07/2020	Tiznit	29D	V	27	P
BS68	F99	02/07/2020	Tiznit	21D	V	29	P
BS69	F100	02/07/2020	Tiznit	13D	V	27	P
BS70	F101	02/07/2020	Tiznit	31D	V	28	P
BS71	F102	02/07/2020	Tiznit	44D	V	30	P
BS72	F103	02/07/2020	Tiznit	36D	V	28	P
BS73	F104	02/07/2020	Tiznit	24D	V	29	P
BS76	F105	02/07/2020	Tiznit	42D	V	30	P
BS77		02/07/2020		42D		28	
BS78		02/07/2020		42D		29	
BS79		02/07/2020		42D		27	
BS80		02/07/2020		42D		30	
BS81		02/07/2020		42D		32	
BS82		02/07/2020		42D		39	
BS83		02/07/2020		42D		39	
BS84		02/07/2020		42D		36	
BS85	F106	09/09/2020	Casablanca	33D	NV	31	P
BS86		09/09/2020		33D		30	
BS87	F107	09/09/2020	Casablanca	27D	NV	30	P
BS88		09/09/2020		27D		30	
BS89		09/09/2020		27D		29	
BS90		09/09/2020		27D		30	
BS91 *	F108	10/09/2020	Rabat	29D	V	24	P
BS92		10/09/2020		29D		29	
BS93		10/09/2020		29D		34	

F: Farm; D: day; V: vaccinated; NV: Non-Vaccinated; P: positive; N: negative; Ct: Cycle Threshold; *: isolated sample; ^+^: sequenced sample.

**Table A2:** HA mutations as compared to 2016 Moroccan strain SF1 (H3 numbering).

	137	188	190	222	226	298	325	364	375	397	402	408	493
SF1	T	D	A	L	L	I	H	M	V	D	E	N	T
AS13	T	D	T	L	L	I	H	I	V	D	E	N	T
AS14	T	D	A	L	L	I	Q	M	V	D	E	N	I
AS29	T	D	V	L	L	I	H	M	V	N	E	N	T
AS32	T	D	A	L	L	V	H	M	V	D	D	N	T
AS71	T	D	A	L	L	V	H	M	I	D	E	S	T
AS76	T	N	A	L	Q	V	H	M	V	D	E	N	T
AS77	T	D	A	L	Q	V	H	M	V	D	E	N	T

**Table A3:** NA mutations as compared to 2016 Moroccan strain SF1 (N2 numbering).

	8	16	31	42	46	50	56	57	58	60	65	80	88	101	116	127	254	261	290	329	332	368	385	390	400	416
SF1	I	T	T	Y	S	A	I	I	I	R	I	T	S	S	V	S	I	K	V	N	S	K	T	S	N	I
AS13	I	T	T	Y	S	A	I	I	I	R	I	T	S	S	V	S	I	R	V	D	S	K	N	A	S	I
AS14	I	T	T	Y	S	T	T	T	T	R	I	P	L	S	V	N	V	K	V	N	S	T	T	S	N	M
AS29	I	T	T	H	P	A	I	I	I	K	I	T	S	S	V	S	I	R	V	N	S	K	T	A	S	I
AS32	I	I	T	Y	S	A	I	I	I	R	T	T	S	S	I	S	I	K	V	N	S	K	T	S	S	I
AS71	M	I	T	Y	S	A	I	I	I	R	T	T	S	A	V	S	I	K	A	N	F	K	T	S	S	I
AS76	M	I	T	Y	S	A	I	I	I	R	T	T	S	A	V	S	I	K	V	N	F	K	T	S	S	I
AS77	I	I	M	Y	S	A	I	I	I	R	T	T	S	S	I	S	I	K	V	N	S	K	T	S	S	I
